# Understanding the relationships between physiological and psychosocial stress, cortisol and cognition

**DOI:** 10.3389/fendo.2023.1085950

**Published:** 2023-03-06

**Authors:** Katharine Ann James, Juliet Ilena Stromin, Nina Steenkamp, Marc Irwin Combrinck

**Affiliations:** ^1^ Applied Cognitive Science and Experimental Neuropsychology Team (ACSENT) Laboratory, Department of Psychology, University of Cape Town, Cape Town, South Africa; ^2^ Division of Geriatric Medicine, Department of Medicine, University of Cape Town, Cape Town, South Africa

**Keywords:** cognition, cortisol, endocrinologist, hypothalamic - pituitary - adrenal axis, glucocorticoids, physiological stress, psychosocial stress, mental health

## Abstract

Stress is viewed as a state of real or perceived threat to homeostasis, the management of which involves the endocrine, nervous, and immune systems. These systems work independently and interactively as part of the stress response. The scientific stress literature, which spans both animal and human studies, contains heterogeneous findings about the effects of stress on the brain and the body. This review seeks to summarise and integrate literature on the relationships between these systems, examining particularly the roles of physiological and psychosocial stress, the stress hormone cortisol, as controlled by the hypothalamic-pituitary-adrenal (HPA) axis, and the effects of stress on cognitive functioning. Health conditions related to impaired HPA axis functioning and their associated neuropsychiatric symptoms will also be considered. Lastly, this review will provide suggestions of clinical applicability for endocrinologists who are uniquely placed to measure outcomes related to endocrine, nervous and immune system functioning and identify areas of intervention.

## Understanding physiological and psychological stress

### Definitions of stress

Intuitively, we know what stress is, and we know that stress is bad for us. However, the current scientific literature contains varying explanations of this construct, which has led to ongoing debates about the effects of stress on the mind and the body (1–5). A useful starting point, therefore, would be to go back to the early definitions of stress.

Hans Selye, a pioneering physician and endocrinologist known in many circles as the founder of stress theory, defined stress as “the state manifested by a specific syndrome which consists of all the nonspecifically-induced changes within a biologic system” (6, p. 64). Hence, he suggested that stress has its own constitution, but without one particular cause, that produces visible changes in response to stress. Selye also wrote about a stress condition known as the General Adaptation Syndrome (GAS). Selye’s view of this syndrome, which he regarded as a form of defence similar to immunity, was that it represented the attempts of an organism to adapt itself to new conditions; he also clarified the role of the adrenal glands in adaptive reactions (7) (the role of the adrenal glands in the stress response will be discussed more in the later section on the hypothalamic-pituitary-adrenal axis). Selye described the stereotypical response pattern that occurs in situations of stress. Stated simply, this pattern included an initial shock phase and an alarm reaction, followed by adaptation to the stressor, and then an exhaustion phase, where persistent aversive stimulation can overwhelm an organism’s ability to resist the stressor (6). Additionally, Selye identified two categories of stress; namely distress and eustress (8). The former refers to stress that leads to one feeling overwhelmed and has a negative impact, and the latter meaning good stress, or stress that has a positive effect. Eustress, which can be brought on by facing an enjoyable challenge, involves a moderate amount of stress with beneficial outcomes. It has the ability to energize and motivate us to overcome illness and obstacles and produce positive feelings of excitement, fulfilment and satisfaction. These categories inform the concept that not all stress is experienced in the same way, which adds to the complexity of understanding varying definitions of stress and its impact on physical and mental health.

Amid these complexities, however, there is general consensus in the neuroscientific literature, that stress can be considered as a stimulus, a reaction to a stimulus, or the physiological effects of that reaction (9, 10). Within this explanatory framework, the neurobiological stress response occurs when the organism is exposed to stressors (i.e., life experiences that threaten a primary goal). By this definition, stressors are categorised as being either physiological (i.e., presenting a threat to one’s physical integrity) or psychological (i.e., presenting a threat to one’s mental well-being) in nature (11, 12). Stressors can be acute or chronic in presentation. An acute stressor (e.g., having a near-miss motor vehicle accident) triggers an immediate stress response that usually subsides shortly after the stressor itself has ceased to exist or is no longer present in the individual’s life. On the other hand, a chronic stressor (e.g., ongoing financial problems or a lengthy illness) occurs over a prolonged period of time, where a simple or quick solution is not available (13). Both acute and chronic stressors may lead to a range of physiological and psychological impairments (14–16).

### The impact of physiological and psychological stress on physical and mental health

In recent decades, research has sought to elucidate the effects of physiological and psychological stress on physical and mental health. This research has confirmed that the experience of stress can be helpful in circumstances in which immediate reaction to a stimulus is necessary for survival and continued wellbeing (17–19). However, when stress is excessive and prolonged, it can affect physical and mental health negatively.

Regarding the physical effects, exposure to chronically stressful conditions can decrease resistance to the effects of the stressor and take a toll on the body in general (and even, under some circumstances, lead to irreversible physiological damage). Hence, people experiencing prolonged periods of stress are at increased risk of, for instance, digestive and gastrointestinal problems, hypertension, diabetes, cardiovascular disease, loss of bone minerals, immunosuppression, and asthma (20–22). In one example of a typical cross-sectional study in this literature, Almuneef et al. (23) used questionnaire-based methods to measure associations between adverse childhood events (ACEs; for example, physical neglect, sexual abuse, exposure to household dysfunction) and chronic disease in a Saudi Arabian sample of 931 adults aged 18 years and older. They found significant associations between self-reports of ACES and those of chronic diseases such as diabetes, hypertension, and respiratory illness. Participants who reported exposure to ≥ 4 ACES had a 2–11 times greater risk of developing a chronic disease during adulthood. Longitudinal studies have confirmed these associations. For instance, Harris et al. (24) explored data from the Australian Longitudinal Study on Women’s Health (*N* = 12,844) and found that moderate-to-high levels of perceived stress in middle-aged adults were associated with a two-fold increase in risk of diabetes diagnosis 3 years after initial reporting of perceived stress (see also, 25). It is recognised that the effects of stress can have significantly detrimental effects on several biological systems (the cardiovascular system being a major one). However, it is beyond the scope of this review to provide more details on the links between stress and all of the biological systems affected.

The experience of chronic stress is also associated with negative mental health outcomes. There is a well-established link between prolonged exposure to psychosocial stress and risk for major depressive disorder (MDD; 26, 27). Animal models indicate that different sources of stress (e.g., learned helplessness, social defeat and social isolation) can alter brain structure and function, thus causing behaviours typically associated with depression (for a review see 28–30). For instance, Peric et al. (31) found that chronic social isolation stress affected the down-regulation of proteins involved in mitochondrial transport and energy processes in the rat hippocampus, causing decreased sucrose preference (a classic marker of anhedonia). Treatment with the antidepressant fluoxetine modified this behaviour, with rats’ subsequent intake of sucrose approaching that of non-stressed controls. Human studies have reported similar patterns of data, particularly with respect to early childhood adversity (32, 33). For instance, Mall et al. (34) found that childhood experiences of bullying, emotional neglect and emotional abuse, as well as recent relationship and academic stressors, were significant predictors of past-year depression in a sample of first-year university students (N= 686).

There are also well established associations between stress and neurodegenerative disease. It appears that the experience of chronic stress increases the risk of developing dementia and that individuals who experience cognitive decline in later life are more likely to have been exposed to chronically stressful events (35–37). Rodent models of Alzheimer’s disease (AD) have been particularly useful in delineating putative mechanisms underlying the relationship between stress and dementia (38–40). For instance, Han et al. (41) reported that chronic stress applied over 4 weeks led to impaired cognitive function (as measured by Morris Water Maze performance) and increased beta-amyloid (Aβ) deposition in APP/PS1 mice, and that these impairments and increases were related to particular metabolic changes (e.g., among amino acids and ketone bodies). In older adult humans, there are significant associations between experiences of stress and impaired cognitive functioning. For instance, Koyanagi et al. (42) investigated associations between stress (as measured by questions about inability to cope with or control everyday events or activities) and clinically diagnosed mild cognitive impairment (MCI) in six low-and-middle-income countries (China, Ghana, India, Mexico, Russia, and South Africa; N= 32715; age= ≥50 years). Those with higher levels of perceived stress had an increased risk of MCI. Similarly, James et al. (43) demonstrated that older adults with mild-to-moderate AD reported significantly higher levels of current psychosocial stress than healthy age-matched controls, and that perceived stress levels were significantly correlated with performance on a standardised episodic memory test.

Finally, large-scale epidemiological studies also suggest that exposure to various kinds of stress (e.g., everyday life stress, work stress, traumatic events and major life stressors) may increase the risk for developing dementia. For instance, Johansson et al. (44) investigated relations between self-reported stress at three time points during mid-life and dementia diagnosis at 35-year follow-up (N= 1462 women; age range at initial assessment = 38- 60 years). Relative to those who reported no significant period of everyday stress during mid-life, those who reported work, health and/or family stressors were 1.10-2.51 times more likely to be diagnosed with dementia at follow-up (with a higher risk of dementia being associated with more consistent reporting of lifetime stressful experiences).

### Moderators of stress effects on physical and mental health

Individual factors can moderate the effects of stress on an organism’s functioning. One such factor is resilience. Resilience is viewed as the maintenance of physical and psychological health in the face of threat and/or adversity (45, 46). Not every person will experience a particular stressor in the same way. A key factor in this is the causal role of stimulus appraisal; that is to say, how one evaluates and interprets a situation or event with respect to one’s wellbeing (47). Research has indicated that differences in psychological resilience account for meaningful variation in daily emotional responses to stress. Further, it has been shown that the experience of positive emotions aids high-resilient individuals in their ability to recover effectively from daily stress (48).

Resilience has also been associated with other important factors that moderate the stress effect, namely age and sex. For instance, older adults seem to possess higher levels of resilience than younger adults, and females tend to have higher levels of resilience and access more social support than males do; however, females have increased vulnerability to stress-induced ailments (49, 50). Aside from their associations with resilience, both age and sex have been identified as independent and joint moderators of the stress response.

With regard to age, the animal literature indicates that advancing age is associated with a decreased stress response (see e.g., 51, 52). Using a longitudinal design, Lendvai et al. (53) measured the stress response of a sample of house sparrows (*Passer domesticus;* age-range = 1-8 years) twice in consecutive years. They found that the birds’ response to a standardised stressor decreased in magnitude as they increased in age. Human literature has delivered less consistent findings, with some studies finding no age effects on stress responses, others finding that stress responses decline with age, and still others reporting that the stress response increased with age (see review on stress and resilience in older adults; 54). One indication of this complexity in the literature is provided by Scott et al. (55), who found that, in contrast to older adults, the immediate responses to everyday stressors of younger adults contained stronger elements of negative affect. However, there were no age differences in responses to those stressors 3–6 hours later.

With regard to sex, animal studies have investigated biological factors that underlie and explain a greater vulnerability of females to stress-related disorders (e.g., MDD and post-traumatic stress disorder, PTSD; 56). For instance, Weisbrod et al. (57) reported that two forms of stress (chronic unpredictable mild stress administered at least once a day for two weeks and shock stress administered for 2 hours on 3 consecutive days) altered certain behavioural (open field activity) and biological (body weight) indicators of depression in female, but not male, Sprague-Dawley rats. In humans, analyses of physiological and emotional responses to stress indicate that women may have a greater stress response than men (58, 59). Data combined from three national surveys analysing psychological stress at three time points (1983, 2006, and 2009) indicated higher levels of stress in women than in men at all time points (60). Likewise, Matud (61) found that women scored significantly higher than men on measures of chronic stress and minor daily stressors, even after adjusting for sociodemographic variables. Further, the lifetime risk for depression and for PTSD in women is twice that of men (62, 63). Although some of this discrepancy in diagnostic rates might be attributed to socio-cultural factors and gender- informed perceptions of illness, it appears that there are credible biological reasons underlying greater female vulnerability to the negative effects of stress. For instance, Herbison et al. (64) found that, for women, medium to high chronic stress exposure or exposure during puberty/adolescence predicted depression and anxiety symptoms, while low or reduced stress exposure during the life course did not. In contrast, for men, prenatal stress (as reported by their parents/caregivers) was a strong and independent contributor to later-life depression, notwithstanding any experiences of postnatal stress.

Implicit in much of the reviewed literature is that the magnitude of stress effects is, to a great degree, dependent on *when* stress occurs (i.e., at what point in the organism’s lifespan the stress is experienced). Exposure to stress during developmentally critical or sensitive periods of early life increases the risk for developing poor physical and mental health outcomes (see, e.g., 65–68). For instance, Schalinski et al. (69) reported that, among a number of different forms of childhood trauma and ACEs experienced by a sample of young adults (*N* = 180, mean age = 28.6 years), (a) the experience of emotional and physical neglect at age 10 years bore a strong relationship to severity of positive psychotic symptoms, and (b) the experience of physical and/or sexual abuse at age 12 years bore a strong relationship to severity of negative psychotic symptoms.

A large proportion of the stress-related literature has focused on understanding the physiological nature of stress and how the brain and body interact to respond to and cope with exposure to a stressor.

## Cortisol and the hypothalamic-pituitary-adrenal axis

There are two neuroendocrine systems that play a significant role in responding to and coping with stressful conditions: the sympathetic adrenal medullary system (SAM) and the hypothalamic-pituitary-adrenal (HPA) axis (the main stress response system of interest in this review).

**Figure 1:**
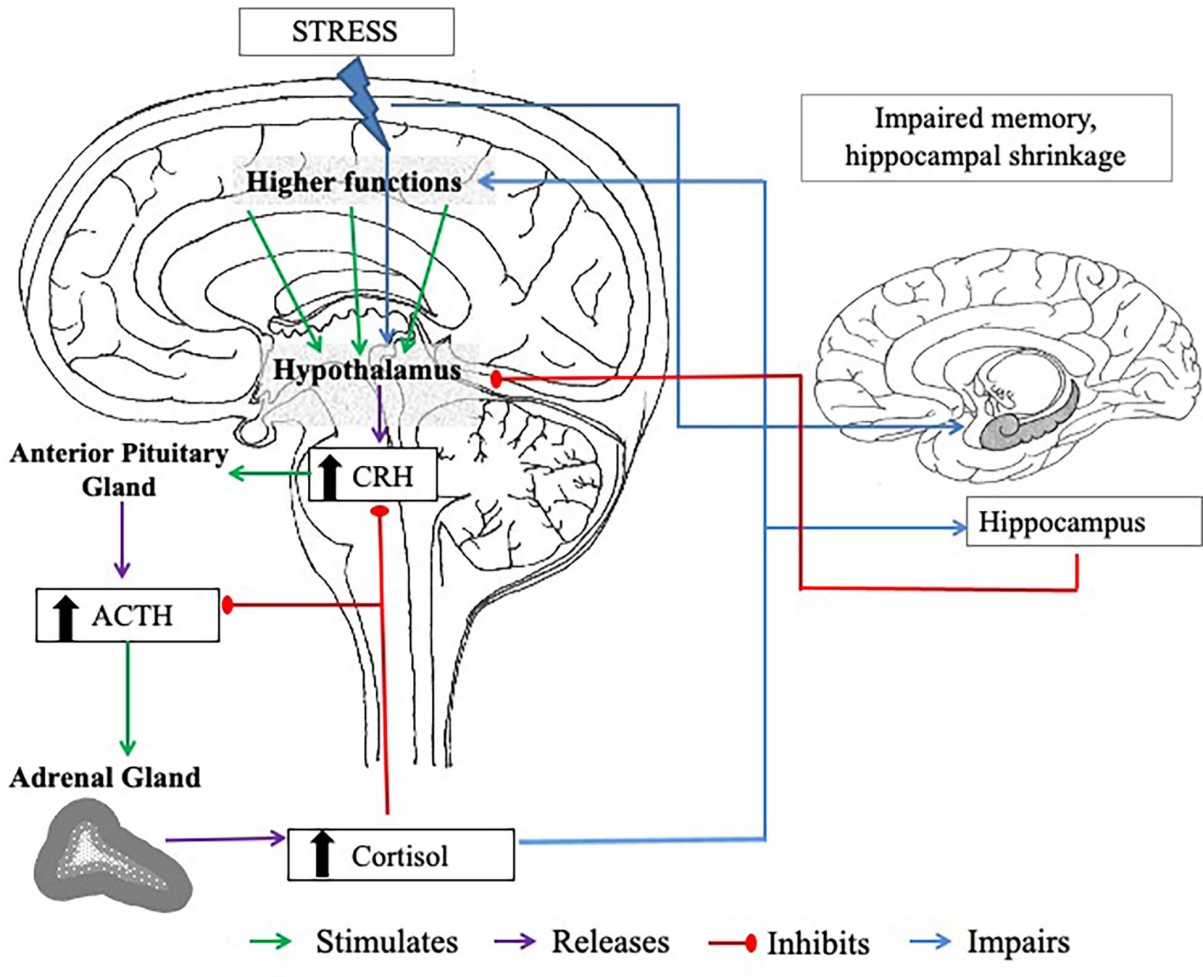
The negative feedback loop of the HPA axis. CRH = corticotropin-releasing hormone. ACTH = adrenocorticotropic hormone. The body experiences a stressor; this triggers the hypothalamus to release CRH, which stimulates the anterior lobe of the pituitary gland to release ACTH. The adrenal cortex of the adrenal glands responds to stimulation by the ACTH and produces cortisol. High plasma cortisol stimulates the hippocampus (a brain region involved in memory processes, that has an abundance of corticosteroid receptor sites), which also has inhibitory control over the HPA axis to prevent excess cortisol release. When stress is chronic, high levels of circulating cortisol can cause long-term damage to the hippocampus, including hippocampal shrinkage, which can impair the actions of the negative feedback loop. Cortisol also has a negative feedback on the hypothalamus and the pituitary gland, inhibiting CRH and ACTH secretion.

In the event of experiencing acute stress, the initial response to this is facilitated *via* the SAM, which regulates the release of catecholamines (including noradrenaline, adrenaline, and small amounts of dopamine) and ultimately triggers the “fight or flight” response (70, 71). These processes lead to activation of the HPA axis. With activation of the HPA axis, the hypothalamus and anterior pituitary are triggered to secrete corticotropin-releasing hormone (CRH) and produce adrenocorticotropic hormone (ACTH), respectively. These processes stimulate the zona fasciculata of the adrenal cortex to release glucocorticoids, of which cortisol is the principal human glucocorticoid (in rodents, this is corticosterone), into the bloodstream (11, 72). The release of this hormone is the best characterised marker of the HPA axis response to psychosocial stress. Cortisol is regulated by a negative feedback system, involving the hippocampus, in which circulating glucocorticoids down-regulate CRH (73) and ACTH (74) secretions from the anterior pituitary and hypothalamus, respectively (see **Figure 1**.).

It should also be noted that cortisol is not the only steroid released from the adrenal cortex; sex hormones (androgens and oestrogens) and mineralocorticoids (the most important of which is aldosterone) are also secreted from the adrenal glands. More specifically related to the stress response, in addition to the release of cortisol, is the release of dehydroepiandrosterone (DHEA) and its sulphated form, DHEA-S. DHEA is understood as a precursor to the production of sex hormones (testosterone, oestrogen, progesterone) but it is also produced in greater quantities as a moderator of the stress response (75, 76). Interestingly, the DHEA-to-cortisol ratio has been associated with increased stress tolerance; in other words, individuals who have a higher ratio (DHEA levels are higher than cortisol levels), seem to tolerate stress better and experience less negative effects from the same stressors as those with a lower ratio.

Decades of research has contributed to the understanding that disruption of the HPA axis can result in dysregulated stress response phenotypes that demand a physiological cost that is referred to as allostatic load. Organisms that experience a high allostatic load may be at increased risk of further challenges. This was demonstrated in a model of non-invasive chronic corticosterone treatment in mice. Results from that study indicated that dysregulation of the HPA axis led to incongruity between the hormonal and neural response to acute stress; this resulted in abnormal behavioural coping strategies that would have negatively impacted on their chance of survival (77). Further, cortisol (corticosterone in animals) augments gluconeogenesis, suppresses immune response, and increases the metabolism of fat and protein. Circadian variation in the HPA axis is the driving force in the overall regulation of adrenal glucocorticoid secretion.

Circadian rhythms are endogenous processes with a periodicity of approximately 24 hours. Circadian rhythmicity stimulates anticipation of repetitive daily events that occur at approximately the same time of day. This process enables the upregulation of most of the major physiological systems in mammals. Cortisol is a foundational element of human physiology and it is present even during foetal life in healthy pregnancies (78, 79). It has been suggested that the foetus is a peripheral oscillator within the maternal system where the peripheral circadian clocks are largely controlled by the maternal circadian rhythm. After birth, a crucial transition takes place involving postnatal integration of the dispersed foetal circadian clocks, that will then imitate the adult-like circadian rhythmicity that is controlled by the suprachiasmatic nucleus of the hypothalamus (80). It is understood that this circadian rhythm develops within the first six months of life; recent evidence suggests that this rhythm is sometimes established as early as one month of age in healthy, full-term infants (81, 82).

Along with melatonin, cortisol is the most widely used biological phase marker to study circadian rhythm; these hormones are both highly rhythmic (83). Cortisol levels peak in the latter half of the night and are highest in the early morning; this is known as the cortisol awakening response (CAR). The CAR usually lasts for about 30-45 minutes immediately after waking. Thereafter, cortisol levels decrease throughout the day, to half of its peak value in the afternoon, with the lowest production at midnight (84).

### Measurements of cortisol

Cortisol has been studied extensively and is an important factor in psychosocial, physiological, developmental, clinical, experimental, and behavioural studies. What is clear from reviewing the literature is that there are a variety of ways in which cortisol is obtained and measured, and this, in part, contributes to some of the challenges of direct comparisons between studies.

Cortisol has been examined *via* several different methods using a range of substrates including blood (serum or plasma), cerebrospinal fluid (CSF; 85, 86), urine (87), sweat (88, 89), interstitial fluid (90), hair (91), fingernails (92), and most prominently, saliva (25).

In the blood, cortisol exists in two forms: the majority of cortisol is bound to carrier proteins, and a smaller portion exists in a soluble free form. When choosing to study cortisol, it is imperative to consider the varying cortisol fractions, the biological functions of their binding proteins, and the relationship to the HPA axis. Measuring cortisol in serum is useful in the diagnosis of conditions such as hypercortisolism and adrenal insufficiency. However, many factors can affect serum cortisol concentration and particularly episodic cortisol secretion. This, therefore, makes the interpretation of a single cortisol value questionable, at best, and hazardous, at worst. Salivary measurements of cortisol may provide some advantages over serum measurements, including providing a more appropriate measure of adrenal cortical function (93).

Salivary measurements of cortisol are favoured due to their simple, quick, and non-invasive technique. These factors also make the measurement of salivary cortisol in infants and children an easier process (94). Long-term storage of saliva samples of cortisol at room temperature has been found to be detrimental to the integrity of the samples, thus freezing is recommended (95). Salivary cortisol samples have been shown to withstand repeated freezing and thawing of samples up to four times before analysis, with no effect on the cortisol concentrations. Further, centrifuged saliva samples of cortisol can be stored at 5°C for up to 3 months or at −20°C or −80°C for at least one year. However, measuring cortisol concentrations in saliva is not without its complications. Salivary cortisol concentrations can be affected by several factors including body weight and body mass (96), daily rhythm (97), caffeine ingestion (98, 99), alcohol consumption (100), antibiotic intake (101), and recent infection (97). Ideally, these factors should be controlled to improve reliability and accuracy of cortisol readings.

### Age and sex differences in cortisol

Salivary cortisol levels have been measured across the age range, including in infants, children, adolescents, and adults. Kiess et al. (96) measured salivary cortisol in samples from all these groups (138 healthy infants, children and adolescents and 14 adults). They found that cortisol levels were age-dependent. Other interesting findings to emerge from their study demonstrated that the highest cortisol levels were measured in saliva of children under the age of 1 year, with no circadian variation before the age of 9 months. They also reported that circadian rhythmicity of salivary cortisol levels only emerged after 1 year of age, and they observed a correlation of cortisol levels after the age of 6 years with pubertal stages. More recent evidence from a prospective longitudinal study of 24-hour urinary free cortisol excretion, in people aged 20 to 90 years old, highlights important questions about the impact of age-related changes in cortisol in predicting medical, physiological and behavioural changes. Findings from that study showed that 24-hour urinary free cortisol to creatinine ratio (UFC/Cr) followed a U-shaped pattern across the life span. This pattern indicated a decrease in the second and third decades of life, relative stability in people in their 40s and 50s, and increases thereafter (102). The impact of age on cortisol levels is clearly an important consideration.

In normal ageing, several endocrine changes occur, including those linked with changes in the structure and function of the adrenal gland. Such changes are associated with alterations in hormonal output, such as gradual sustained elevation in glucocorticoid secretion (although a similar alteration in normal circadian rhythm has not been observed; 103). Veldhuis et al. (104) described the relationship between ACTH and rising cortisol levels as humans age; this was confirmed by Roelfsema et al. (105), who demonstrated how increasing age was linked with elevated cortisol concentrations in the late evening and early night and advanced the timings of the peak diurnal rhythm. These findings correspond with earlier studies that reported significant associations between older age and higher baseline cortisol (106, 107). Furthermore, the capacity to secrete DHEA and DHEA-S in response to acute stress declines with age; therefore reducing the potential beneficial effect of the DHEA-cortisol ratio discussed earlier in this article (76, 103).

The majority of studies on cortisol have included smaller, selected samples which have limited generalisability of the findings. Larsson et al. (107) aimed to address this in a cross-sectional study of 1811 men and women (30-75 years of age) that specifically investigated age differences in diurnal cortisol patterns. They found that in males and females, there was a significant association between age and increased cortisol on all measures (this association was most consistent with evening cortisol). The elevation of cortisol levels in older adults is of particular relevance due to the effect of cortisol on several systems including cognition, and the known relationships between chronic stress, raised cortisol levels and ageing. Furthermore, excessive cortisol levels can lead to changes in the structural and functional integrity of several important brain regions such as the hippocampus, amygdala, and prefrontal cortex (108). The subsequent impact on cognitive functioning as a result of these changes will be discussed in more detail in later sections of this article. It is also pertinent to note the impact of chronically high cortisol levels on the stress response in older adults, which can result in impaired ability to recover from stressful stimuli (103).

In addition to age differences, several studies report sex differences in cortisol levels (105, 107, 109). These largely exist due to biological sex differences in the HPA axis itself, which leads to sex-dependent responses to stress. Further, sex steroids are considered to play an important modulatory role in the sex-differentiated stress response (110, 111). For instance, testosterone has been negatively correlated with salivary cortisol in men, while progesterone was negatively correlated with cortisol responses in women, suggesting inhibitory functions of these steroids in men and women, respectively (112). Furthermore, cortisol levels in older women in response to stress have been found to be higher than in males (106). Interestingly, and supported by findings from Seeman et al. (106) and Hidalgo et al. (113), younger women appear to produce lower levels of cortisol in response to stress than young men. This finding is contradicted by Batabyal et al. (114) who reported that in a sample of university-age students, men showed significantly lower levels of salivary cortisol than women over the period of one year. Despite these varying results, there is evidence to suggest significant age-by-sex interactions regarding patterns of stress response and cortisol secretion (106, 115).

As inferred from the above discussion, the measurement of cortisol is complex and affected by many factors. Single-point measurements are not that helpful and cortisol assessments are only performed in laboratories which also make them less accessible. Given that cortisol levels can provide indicators of HPA dysfunction and the presence of underlying, potentially treatable conditions, it would be helpful to be able to measure cortisol more efficiently. Improved accuracy and faster detection of cortisol may have significant implications for preventing, diagnosing and treating stress-related disorders, as well as for those experiencing adrenal insufficiencies. Recent investigations point to technological advances and the development of promising proto-types, including wearable devices with electrochemical sensors (89, 116). Future developments in this vein may lead to the provision of rapid, accurate, and repeated cortisol assessment in everyday life. It would be premature to over-interpret this information at this stage but it is something to bear in mind for the future.

Cortisol is known to permeate the blood brain barrier (3). Research shows that this is particularly true when the stressor persists longer than 15-60 minutes. In the context of acute stress, glucocorticoids interact with corticosteroid receptors (mineralocorticoid and glucocorticoid receptors) located throughout the brain. There is an abundance of both mineralocorticoid and glucocorticoid receptor in the hippocampus, amygdala, and in the prefrontal cortex (117–119). These receptors play a key role in regulating the neural circuits and neuroendocrine systems that instigate behavioural responses to stress (120).

Glucocorticoid receptors play a role in both short- and long-term neuro-biological changes found in response to stressors (121). For example, environmental circumstances that occur early in life can lead to permanent changes in (a) the development of glucocorticoid expression in the hippocampus, and (b) HPA axis responses to chronic stress (122). Further, glucocorticoids can alter the hippocampus in several ways: they can reduce excitability of some hippocampal neurons, they can lead to atrophy of dendritic branches in pyramidal cells within the CA3 area, and they can stunt the growth of new neurons in the dentate gyrus (123). Of note, these areas have been implicated in many cognitive processes including executive functioning, memory, and learning (124). High levels of cortisol are associated with impaired memory; however, ongoing evidence suggests that low elevations of cortisol may also lead to deleterious effects on memory (125, 126).

While the physiological response to acute psychological stress is well documented, the response to prolonged psychological stress is less well known. Žarković et al. (127) designed a prospective assessment of cortisol secretion during prolonged psychological stress that was induced *via* continuous air raids and after cessation of the stressor. Psychological, endocrine and psychiatric assessments were conducted at two months and at 18 months after cessation of the stressor. In their small sample of healthy participants (aged 34-39 years old), they found that prolonged psychological stress was associated with a transient suppression of the HPA axis. This was manifested by low morning cortisol and reduced cortisol response to ACTH. They concluded that the reduced cortisol response was sufficient to result in a false diagnosis of HPA insufficiency. Such findings support the “adrenal fatigue” theory that prolonged exposure to stress can drain the adrenals (due to an inability to keep pace with the demands of an ongoing perceptual fight-or-flight arousal state) leading to a low cortisol state. However, validity of this theory has been disputed and remains unresolved due to a lack of hard evidence to support it (128).

Cortisol depletion subsequent to prolonged or excessive secretion can contribute to its dysfunction, but alternate explanations should also be considered. In addition to depletion of cortisol, cortisol dysfunction may arise from several other neuroendocrine alterations such as impaired cortisol secretion, a deficiency in free (unbound) cortisol, glucocorticoid receptor resistance, or dysregulation of the negative feedback system (129–132). Irrespective of which neuroendocrine mechanism is responsible for ensuing changes, the long-term effect of chronic stress is the same; namely, that cortisol fails to function.

### Apolipoprotein E and the HPA axis

Another factor that appears to influence the HPA axis response to stress and circulating cortisol levels is the є4 variant of apolipoprotein E (APOE-є) (133, 134). APOE-є is a protein that is involved in lipid transport and metabolism. It is produced and secreted in the brain and is involved in neuronal regeneration (135). In humans, the apolipoprotein E phenotype is coded by a gene that has three common allelic variants: є2, є3, є4 (136, 137). Rodents express only one form of APOE-є.

Several animal studies have explored how stress and APOE genotype interact to influence glucocorticoid levels. For example, one study found that elevations in glucocorticoid levels after constraint stress were markedly lower in APOE-deficient mice than in control wild-type mice (138). Findings from another study involving predator stress suggest that the effect of stress on cognition is mediated by corticosterone, and that this effect is modified by APOE presence (139). Further, increased levels of cortisol in APOE-є deficient mice suggest that APOE genotype may influence cortisol concentrations in neurological disorders such as AD (140). Rodent studies have also suggested an important role for APOE-є in regulating adrenal steroidogenesis, and have alluded to the possibility that APOE-є activity may be involved in disorders of glucocorticoid hypersecretion (141).

In humans, a growing field of research has explored the impact of APOE genotype on stress response-related processes, identifying strong links between mitochondrial function, endoplasmic reticulum stress, and the immune response (see mini review by 142). Additionally, carrying the APOE-є4 allele may pose a vulnerability factor in the negative effects of HPA axis dysregulation on cognition during ageing (143). An increased frequency of the APOE-є4 allele has been associated with higher CSF cortisol concentrations in individuals with AD relative to controls (140). It has been suggested that the neurodegenerative process of AD may be initiated by gene-environment interactions that involve APOE-є4 (144–146). For example, Peavy et al. (147) examined the interaction between an environmental factors (i.e., “real-life” stress) and a genetic risk factor for AD (i.e., the є4 allele) in explaining cognitive performance in a sample of 91 non-demented older adults (mean age: 78.8 years). Low-stress participants demonstrated better cognitive performance than high-stress participants on tests of delayed recall, list learning, and visual memory. Participants without the є4 allele obtained better scores than participants with є4 on tests of immediate and delayed recall of visual designs. The authors also reported significant stress by APOE-є4 interaction effects on memory and cortisol in the high-stress, є4-positive group. Those participants displayed consistently poorer memory performance compared to the high stress, є4-negative group, the low-stress, є4-positive group, and the low-stress, є4-negative group. Participants in this group also had higher cortisol levels than those in the low-stress, є4-positive group; cortisol levels did not differ in the high- and low-stress groups who were є4-negative. Similarly, Lee et al. (137) investigated APOE-є4 carrier status, cortisol levels, and cognitive function in community-dwelling older adults. Their results showed that, even though a higher cortisol level was associated with lower cognitive scores, the slopes of the adverse relations were steeper in individuals with at least one APOE-є4 allele. The authors suggested that cortisol’s relationship with cognitive functioning is modified by APOE-є4, such that the є4 allele increases the vulnerability of the ageing brain to the negative effects of stress.

### Psychological and neuropsychiatric implications of HPA axis alterations

It is well understood that the health of the body and mind are inextricably linked; the most well established example of this is the association between psychological stress and psychological ill-health. There is a significant body of existing and ongoing evidence that suggests a link between HPA axis dysregulation and the risk of developing psychiatric disorders, including depression, schizophrenia, and anxiety disorders (148–150). While it is beyond the scope of this review to report comprehensively on the associations between HPA axis functioning and mental health disorders, it is salient to provide a brief overview of the more general psychological implications of HPA axis alterations.

Chronic stress plays a significant role in the development of depressive disorders and it is associated with elevated cortisol levels. Individuals with MDD have significantly higher stress and cortisol levels when compared with controls (151). Associations have been demonstrated between cortisol hypersecretion and both acute and more severe subtypes of MDD (29). The association between increased cortisol levels and the development of depression may relate to excessive adrenal activity acting destructively on the hippocampus and increasing one’s vulnerability to depression (152, 153). Despite known associations between cortisol and MDD, the current general consensus appears to remain uncertain and cautious about the value of assessing cortisol as a biological guideline for the pathophysiology or treatment of MDD. Furthermore, the absolute level of cortisol is viewed as unreliable in predicting the efficacy of antidepressant treatment.

To further complicate matters, questions have been raised about the linearity of the relationship between stress and mental health disorders, such as depression. The early stress literature examining the links between stress and depression was largely based on the tacit assumption of a unidirectional relationship (the stress model of depression: where the experience of stress was assumed to increase susceptibility to depression). However, a comprehensive review in this area (154) concluded that later stress research supported the role of depression in predicting generated stress (the stress generation model; 155); further research is still required in this domain. Recent evidence has pointed to genetic variations in the HPA axis functioning as moderating the effects of psychosocial susceptibility markers on the development of proximal life events (156).

It is salient to note that the psychological determinants of an individual’s response to stress are important predictors of stress-related outcomes. Not all people will experience a stressor in the same way subjectively or experience an endocrine response to a stressor; what may be a stressful experience for one person may be a non-event for another. Exploring endocrine stress reactivity, Fischer et al. (157) measured subjective perceptions of stress and salivary cortisol levels in nurses and physicians working in neonatal and paediatric critical care environments. They found that even though there were frequent stress-related cortisol surges in these participants, the cortisol increases were independent of subjective stress perception and professional experience did not reduce stress reactivity. Additionally, from a biological perspective, the release of cortisol in response to a stressor may attenuate the unpleasant feelings associated with the stressor and ultimately provide beneficial outcomes. For example, small doses of cortisol release in healthy individuals can improve memory and motivation, enhance one’s immune system and increased pain threshold (158–160). Some researchers have reported associations between stress-induced salivary cortisol level elevations and reduced negative affect (161). Such findings suggest a potential mood-buffering cortisol effect and this contributes to the divergence in the literature seeking to associate stress-induced psychological reactions and cortisol levels. Another challenge in interpreting the impact of stress exposure, is that the majority of daily life stressors that are studied, are relatively mild in biological terms. This raises questions about their detectable influence on cortisol secretions and leads to its own interpretation difficulties (162). An additional factor for consideration is the potential for habituation; whereby individuals repeatedly exposed to mild or moderate stressors often adapt to the stress. Such adaptation consequently leads to a reduced cortisol response (163). Such factors result in interpretation difficulties regarding the relationships between psychological stress and cortisol.

Neuropsychiatric effects have also been reported in conditions of adrenal insufficiency, such as in the case of Addison’s disease. Addison’s disease is a medical condition marked by chronic adrenal insufficiency resulting in a failure of glucocorticoid secretion. It presents with a constellation of primary signs and symptoms including but not limited to: extreme fatigue, weight loss, hyperpigmentation, hypoglycaemia, nausea and muscle or joint pain. Other symptoms can include psychosis, irritability, depression and behavioural symptoms; however, the neuropsychiatric component of the condition is less well understood (164). It is relevant for physicians to be aware of the neuropsychiatric symptoms that can manifest in patients with Addison’s disease, because on occasion, these symptoms may be the only manifestations of a potentially life-threatening Addisonian crisis. Additionally, motivation and behaviour during an Addisonian crisis are two of the more unusual presentations, which can make the diagnostic process more challenging (164, 165).

As a side note, low levels of cortisol have been associated with fibromyalgia, chronic fatigue, chronic pain syndromes, and other functional somatic disorders. However, the relatively moderate cortisol reductions are generally not considered to represent Addisonian insufficiency (166, 167). Lower levels of cortisol may be due to underactivity of the HPA axis, partial primary adrenal insufficiency, increased negative feedback sensitivity and/or altered glucocorticoid metabolism. The majority of research examining lower cortisol levels has been conducted in the context of PTSD, where lower levels of cortisol have been associated with early-life physical or sexual abuse (168, 169). This stands in paradox to a wealth of evidence demonstrating associations between severe stress-related psychiatric disorders and elevated levels of cortisol. PTSD arises from exposure to extremely traumatic experiences but it only occurs in a proportion of people exposed to such events. Suggested explanations for this speak to individual variation in vulnerability and resilience.

### The impact of adrenalectomy surgery on HPA axis functioning and brain and mental health outcomes

Another factor to consider in relation to HPA axis alterations and brain and mental health outcomes, is removal of the adrenal glands. Adrenalectomy surgery involves removing one or both adrenal glands. This is often due to the presence of a tumour (generally benign) that is either large in size or is producing excess hormones, which creates problematic health conditions. The majority of adrenalectomy-related research has been conducted in rodents, exploring a number of outcomes such as gene transcription and expression (170), alterations in hippocampal neuronal activity (171), and psychostimulant sensitivity (172).

In humans, there has been an increase in the prevalence of incidental adrenal masses found on routine imaging, as well as increased rates of adrenal operations (173). The standard treatment procedure for such masses involves hospitalisation and endoscopic adrenalectomy. Evidence suggests that adrenalectomy in the context of lateralised primary aldosteronism has beneficial effects not only in terms treating the biological condition, but also in terms of improving mental health outcomes. For example, Citton et al. (174) reported improved postoperative mental health outcomes as evidenced by scores on the Mental Component Summary and Depression Scale questionnaire; these improvements held at early (one month postoperatively) and late follow-up (at least 6 months postoperatively).

Adrenalectomy surgery might also be performed in the context of conditions such as Cushing’s syndrome (hypercortisolism). This syndrome can occur when the adrenal glands produce too much cortisol and it is a potentially life-threatening condition if untreated. In the case of adrenalectomy, glucocorticoid secretion decreases and this leads to increased neuropeptide expression and secretion by the paraventricular nucleus. These processes can occur in both basal and stress-induced states and can make stressors less well-tolerated (175).

In addition to the presence of several physical symptoms, patients with Cushing’s syndrome experience a range of neuropsychiatric and cognitive symptoms, as well as brain atrophy, indicating involvement of the central nervous system. Cushing (176) provided the first description of psychiatric disturbances in Cushing’s syndrome where he highlighted the presence of “emotional disturbances” as a pathologic feature of this syndrome (177). Further studies have been conducted to better characterise the spectrum and frequency of psychiatric and neurocognitive symptoms linked with Cushing’s syndrome (178, 179). The most important of these clinical symptoms include MDD (prevalence 50-81%), bipolar disorders (prevalence 30%), anxiety (prevalence 66%) (180), and neurocognitive impairment (most frequent reported alterations are memory impairment, approximately 83% of cases, and reduced concentration, 66% of cases) (179). Of these, atypical depression is the most common.

Dorn et al. (181) examined the longitudinal course of psychopathology in Cushing’s syndrome after correction of hypercortisolism in patients with active CS. Before cure, 66.7% of patients had significant psychopathology (mainly atypical depressive disorder). Post-cure, overall psychopathology decreased significantly to 53.6% at 3 months, 36% at 6 months and 24.1% at 12 months. Atypical depressive disorder remained as the prevailing diagnosis and an increased frequency of suicidal ideation and panic was reported.

Brain structural abnormalities associated with Cushing’s syndrome have been found to include smaller hippocampal volumes, enlarged ventricles, and cerebral atrophy (182). The impact on brain volume is partially reversible after correction of hypercortisolism, but still persists to some extent (183). Furthermore, there is evidence to suggest that after long-term remission, patients with Cushing’s disease still experience chronic fatigue (184), reduced quality of life, and impaired cognitive functioning (particularly in the domains of attention, visuospatial orientation, reasoning, working and speed memory, verbal fluency and executive functions (179, 185). Thus, the picture of whether CS remission completely resolves psychiatric and neurocognitive disorders remains uncertain. Such findings speak to the importance of considering the psychological, psychiatric, cognitive and behavioural implications of HPA axis dysfunction and adrenalectomy. While psychopathology generally seems to improve with time post-surgery, healthcare practitioners should remain alert to any changes in psychiatric and cognitive symptomology including changes in mood, suicidal ideation, panic and cognitive functioning. The findings also highlight the importance of follow-up checks and monitoring of symptoms; the management of which should be considered as one of the essential outcomes of CS patients.

## Exploring the relationships between stress, cortisol and cognition

### Stress and cognition

Stress affects cognition in several ways depending on its intensity, timing, duration, and origin (186, 187). The literature, which spans both animal and human studies, differentiates between mild-to-severe stressors, time of stress exposure, acute and chronic exposure to stress, and different origins of stress (physiological or psychological).

### Stress and cognition: Findings from animal studies

Animal studies play an important role in advancing the understanding of numerous medical conditions. There exists a vast literature documenting the responses to and effects of stress in laboratory animals. Rodent studies (including both rats and mice) have been invaluable in the study of stress and stress-related conditions as they provide insight into the effects of stress on mental and physical functioning (188). Humans share approximately 90% of their genes with rodents and there is significant overlap in terms of organ systems and functions performed; this makes mice an effective model for human biology and pathology (189, 190). A common method involves exposing animals to one or more stressors that are uncontrollable and often unpredictable. Such exposures might include isolation, confinement or restraint, noise, and separation from companions. Both acute and chronic stress conditions can be studied in rodents; these specific stressors include the forced swim test, inescapable tail-shock, immobilisation, and cold and ether stress. As with humans, the HPA axis in rodents controls their adaptation to stress *via* the actions of the corticosteroid receptor systems in the CNS. The stress manipulations used in rodent studies tend to increase behaviours that are similar to processes observed in human psychopathology, such as anxiety disorders, depressive illnesses, and PTSD (191).

Rodent studies have also demonstrated that stress exposure can alter important cognitive functions such as learning and memory processes (192). For example, rat pups deprived of early support from their dams later exhibited hippocampal-dependent cognitive impairment (193, 194), as well as marked anxious and depressive behavioural changes and deficits in working memory and attention set-shifting in their adult years (195, 196). Additionally, rats that were separated from their mothers early on in their lives were found to later develop AD-like pathology (197–199). In a similar rodent model of PTSD, rats exposed to single prolonged stress, beginning at 25 days old, equivalent to childhood in humans, showed anxious-like behaviours at day 32 and 60 which later expressed as depression by day 90 (200). Short term memory deficits were also observed at day 32 and 60 but not day 90. In terms of timing, age of stress onset is a mediating factor in animal models worthy of further investigation. Ricon et al. (193) found that onset of stress, in the form of food deprivation, elevated heights and forced swimming, affected learning dependent on whether the rat was between 30-90 or 60-90 days old; learning and memory were negatively affected by adult-onset stress but not by juvenile-onset stress. This suggests that juvenile exposure to stress may induce resilience rather than impairment. This is in contrast to literature indicating that ELS leads to cognitive impairment in later life. It also raises questions about the role of resilience and whether this is an intrinsic or acquired trait.

Sex also appears to be a mediating factor of stress in rat models, where sex hormones (testosterone and oestrogen) play an influential role in HPA axis activity (201). Both acute and chronic stress as measured by electrical foot shock and the paradigm for chronic unpredictable mild stress, respectively, produced differences in plasma sex hormone levels (202). Acute stress decreased these hormone levels in female rats while it produced no effect or increased effects in the male rats. The chronic stress paradigm produced greater changes *via* hypothalamic stress-related molecule expression in female rats than male ones, which was associated with greater depression and anxious-like behaviour. The effect of chronic stress on corticosterone concentrations and corticosteroid-binding globulin further strengthen these findings (203).

Similarly, HPA axis hormone plasma levels, as well as corticosterone concentrations, were significantly increased for female rats as compared to their male counterparts, following an acute period of physical spatial restraint (204). Differences in ACTH concentration were not observed at baseline, suggesting that there is an interaction effect between sex and ACTH concentrations in rats. While females expressed greater corticosterone concentrations before and after restraint, corticosterone differences were significantly emphasised following the stress condition. Similar findings by Figueiredo et al. (205) and Heck and Handa (201) suggest that inhibitory inputs to the HPA axis are reduced in females as compared to males following acute restraint. In contrast to this, rats exposed to 10 consecutive days of loud noise for 30 minutes resulted in no differences in ACTH levels and corticosterone concentrations between female and male rats (206). The variance in the effects of sex on stress responses in the above-stated findings is interesting. It may represent inconclusive findings about the overall effect of sex or it may have occurred due to differences in stress techniques used (e.g. physical restraint versus noise).

Animal studies have also examined the effects of stress on brain regions in rats. Stress has been found to cause atrophy in a number of hippocampal subregions (207, 208), as well as in the thalamus and the visual cortex in rats (209). The chronic stress effect is mediated by length of exposure to the stressor according to one study by Luine et al. (210) who found that exposure lasting from 3-6 weeks caused deleterious effects in rats while exposure lasting 3-10 consecutive days improved explicit memory and spatial learning. Furthermore, there are sex differences in the effects of chronic stress on brain regions in rodents; Lin et al. (211) reported abnormalities in brain morphology (atrophy in CA1, CA2, CA3 and amygdala, anterior cingulate area, dorsal part regions) following chronic stress exposure in male rodents but not in females. Such findings suggest a sexual dimorphism in the molecular response to stress in rodents.

As mentioned earlier, prolonged stress has been identified as a contributing factor to the development of neurodegenerative diseases such as AD. This was demonstrated empirically by Carroll et al. (212) in a restraint/isolation-induced chronic stress model that aggravated the accumulation of Aβ in transgenic mice with an APP mutation (Tg2576). The researchers then used this stress paradigm in a tau transgenic mouse model with the P301S mutation (PS19). The combined findings indicated that prolonged stress, arising from a dysregulated HPA axis, increased the production and deposition of Aβ, as well as tau phosphorylation. Such findings provide support for the hypothesis that prolonged stress may contribute to the neuropathogenesis of AD.

Chronic stress has also been found to downregulate glucocorticoid expression in the prefrontal cortex (PFC), which has been linked to cognitive dysfunction in animal models (213–215). In these same models, dendritic shrinking and the remodelling of cell structures was found within the PFC (187). While reversible, following cessation of a stressor, the formation of the affected dendrites shifted in position to the cell body, possibly affecting cell connectivity and subsequent gene expression (216, 217). Specifically, these findings and subsequent cognitive impairment are comparable with those following a medial PFC lesion (187). Conversely, chronic stress appears to lead to dendritic expansion in the orbitofrontal cortex as well as the basolateral amygdala (218–220).

Based on evidence from animal studies, it is clear that brain regions are affected differentially by exposure to stress. What remains unclear is how variables such as sex, type of stressor, age of organism at time of stressor and duration of stressor impact on the brain and body of the organism. Further, the applicability of findings from animal studies to humans remains a complex task. One of the challenges for future stress-related research will be to identify links between the cellular changes observed in animal models of chronic stress to behavioural effects, and to understand the risks they pose for humans for the precipitation of stress-related disorders.

### Stress and cognition: Findings from human studies

Evidence provided by animal studies conducted over numerous decades has confirmed the significant effect of stress and stress hormones on the brain and body. In recent decades, researchers have explored similar effects in humans, largely through the use of laboratory-induced psychosocial stressors such as the Trier Social Stress Test (109). An important area of such research has focused specifically on the relationship between stress and cognition.

Long-term or chronic stress, particularly in childhood and adolescence, consistently affects cognitive mechanisms. It is widely accepted that experiencing early life stress can result in deleterious effects such as psychiatric disorders, depression, anxiety and PTSD, (213, 221–225). Research has also linked early life stress to dementia, cognitive impairment and chronic inflammation in later life (226). In one systematic review conducted by Seifan et al. (227), AD progression was particularly linked to stress associated with early life socioeconomic status. This finding is further supported by a study investigating the role of childhood trauma in Australian Aboriginals, a historically, socially and economically disadvantaged group. Those results demonstrated a significant correlation between childhood trauma and the development of dementia in later life (228). This same finding was reported in a longitudinal design following two different German cohorts (229).

Most studies exploring the effects of stress and cognition using laboratory-induced stressors and pharmacological approaches have focused on global cognition or basic memory functions such as memory consolidation. For example, Leng et al. (230) in drawing data from the EPIC-Norfolk study, recruited 5129 middle-to-older aged men and women (48-90 years) and found that self-perceived stress and stressful life experiences were negatively correlated with scores of global cognition as measured by the Mini Mental State Examination. However, more recent studies have expanded their research approach to include investigating the impact of stress on other cognitive domains. For example, acute episodes of mild-intensity stress can enhance cognitive functions, particularly tasks such as encoding and memory consolidation of task-relevant stimuli and implicit memory or basic declarative tasks. However, when exposed to high-intensity stress, the ability to form and retrieve explicit memories and cognitive processes involving complex reasoning becomes impaired. Exposure to stress has also been associated with tasks of attention; however, conflicting results have been reported, with some studies reporting a negative effect of mild acute psychological stress on attention (231, 232), and other findings demonstrating improved performance (including response time, vigilance and sensory intake) on attention tasks with exposure to mild acute stress (233, 234). Sandi (186) further linked chronic stress to impaired explicit memory processes which pertain to conscious retrieval of factual information in individuals. These processes are reliant on hippocampal and prefrontal functioning (235). At the same time, chronic stress appears to strengthen implicit memory, information that cannot be declared, which is more reliant on the amygdala and the striatum of the limbic system (186).

### Mitigators and moderators of human stress and its impact on cognition

To complicate the relationship between stress and cognition further, the deleterious effects of psychosocial stress on cognition may be mitigated by certain protective factors. These include high resiliency; individuals with a strong belief in their own abilities, also known as self-efficacy; and those with a strong cognitive reserve (236, 237). The latter refers to the brain’s ability to make use of alternative networks in order to sustain baseline functioning following impairment or changes (238, 239). Cognitive reserve is closely linked to education as well as global cognitive functioning (229). Those with a weak cognitive reserve as well as those with a self-perceived lack of control appear particularly vulnerable to the effects of social stressors on cognitive performance (240, 241).

Education has also been identified as a moderator of the effects of stress on cognition. Individuals with a higher level of education and reportedly greater engagement with cognitively stimulating tasks, even in later life, have reduced risk of developing MCI and AD (229). Moreover, those with a low education level appear more at risk for developing AD in later life (227). Similarly, Tschanz et al. (242), in their population based study, found that the relationship between stressful life events and cognitive functioning was moderated by level of education as well as the amount of stressful life events experienced. Lower levels of education and greater stressful life events appeared to accelerate cognitive decline. This relationship was further moderated by age: younger persons experienced accelerated cognitive decline in relation to a greater number of stressful life events than their older counterparts. However, this study did not account for variables such as stress duration or subjective intensity which have been noted as possible mediators of stress.

Further, age and sex have been found to modulate the relationship between stress and cognitive performance (56, 113, 243). Of note, sex differences in recall performance were only pronounced in a young sample as investigated by Hidalgo et al. (113). The chosen acute stressor (TSST) in that study was significantly and negatively associated with memory performance in men while this relationship was not found amongst the women.

### Stress, cortisol and cognition

Within the stress literature, the majority of studies have explored relationships between psychosocial stress and cognition or between cortisol and cognition. Fewer studies have included measures of both psychosocial stress and cortisol in relation to cognitive functioning. In humans, research has demonstrated positive correlations between psychosocial stress and cortisol levels (241, 244, 245), as well as identifying negative correlations between psychosocial stress and cognitive functioning (43, 241, 244–246).

Recent findings (247) confirm results reported from previous studies (124, 241, 243, 248) demonstrating that an abundant release of glucocorticoids can modulate cognition. Sussams et al. (249) used a multi-centre longitudinal study of individuals with amnestic mild cognitive impairment (aMCI) to explore psychobiological stress and cognitive outcomes. Measures of psychological stress included the Recent Life Changes Questionnaire (RLCQ), the Perceived Stress Scale (PSS) and salivary cortisol. Scores were examined in relation to rates of cognitive decline over an 18 month follow-up period and conversion to dementia over 5.5 years. They found that, compared with controls, PSS scores were higher in the aMCI group, but there were no between-group differences in RLCQ scores or salivary cortisol measures. RLCQ scores and poorer cognitive function at baseline were associated with high salivary cortisol levels but no relationship was found between salivary cortisol and conversion rate to dementia. The authors proposed that psychological stress (as measured by the RLCQ or PSS) was not associated with adverse cognitive outcomes in an aMCI group. Instead, they postulated that this may be an indication of diminished cortisol production to psychological stress with disease progression.

Similar results were reported by another longitudinal study conducted by Ouanes et al. (250), investigating cognitively healthy older adults, which suggested that significantly high salivary cortisol levels were not produced on account of stressful life events. While stressful life events do appear to be predictive of developing dementia in later life, it is not yet clear whether stressful life events are directly correlated with poor cognitive functioning (242). Indeed, recent literature links high levels of cortisol to poor cognitive performance only (251–253). However, in the longitudinal study conducted by Sussams et al. (249), both self-report questionnaires and salivary cortisol levels were not predictive of conversion to dementia over the 5.5 year period.

Regarding acute stress, different tasks elicit different stress responses in humans (186). For example, physical stressors, such as cold water submersion and mental tasks that induce cognitive stress, trigger the sympathetic nervous system while psychosocial tasks are associated with activation of the HPA axis (71, 113).

Factors that appear to mediate the relationship between memory functioning and cortisol include the point at which memory is tested (i.e., during encoding or retrieval, etc.), level of arousal, the amount of cortisol reported, and the specific brain region upon which the memory function is dependent (43, 186, 254). Moreover, the time course of stress hormones in the human body may be responsible for memory functioning: when levels of both catecholamines and glucocorticoids are elevated, individuals are able to effectively encode and consolidate information relevant to the stressor. Information that is not relevant to the stressor is neglected and impaired. Consequently, when catecholamine levels have dropped but glucocorticoid levels remain high, consolidation processes are optimised at the expense of encoding and retrieval (186, 207, 255). It is further posited that there is a window wherein memory may be restored given normal glucocorticoid levels which restore the protective brain derived neurotrophic factor (256).

Some studies have found a dose-dependent inverted U-shaped relationship between cortisol and memory performance during retrieval (187, 247, 257). One study found this effect after administering cortisol equivalent to 0, 3, 6, 12, and 24 mg intravenously (257). Both Schilling et al. (257) and Wu and Yan (258) demonstrated how rapidly these acute doses of cortisol impair retrieval in healthy individuals (approximately 8 minutes). Other research suggests that age may be a mediating factor in which younger people are less vulnerable to the effects of cortisol on memory performance than their older counterparts (43, 259–262). These findings might elucidate the negative effect of chronically high levels of cortisol on cognitive functioning found in individuals with AD (246).

In the context of older adults, and similar to findings from animal studies, in cognitively healthy older adults, Aβ has been associated with increased cognitive decline. Further, elevated plasma cortisol levels may expedite the Aβ effect on decline in global cognition, episodic memory, and executive function (263). Further, in a study investigating 28 young adults and 32 older adults in two different conditions (a familiar learning environment for each age group and an unfamiliar learning environment for each group), older adults performed significantly worse on tasks of delayed recall and had higher cortisol levels during the unfamiliar condition (260). This relationship was inverted when older adults were tested within the familiar condition for their age group. The young adults were not significantly affected by the familiarity of the condition for both memory performance and cortisol levels. In contrast, scores on immediate recall did not appear to be affected by the condition or the cortisol levels recorded. Researchers of this same study suggested that the variation in performance between delayed and immediate recall might be due to the role of the hippocampus in delayed recall processes and the abundant supply of glucocorticoid receptors in this same brain region. This would render the hippocampus more susceptible to elevations of cortisol levels and would subsequently impair delayed recall. Overall, across conditions and despite differences in cortisol levels, the young adults performed significantly better than their older counterparts. However, the magnitude of these age-related differences might be mediated by the stress of the environment. Further, in this sample, basal salivary cortisol levels did not differ between the young and older adults.

Executive functioning performance has consistently been associated with cortisol levels, stress, and the HPA axis in humans (235, 264, 265). High cortisol awakening response (cortisol measured during the first 30 minutes following sleep) has been associated with executive dysfunction (266). It is hypothesised that because executive functioning is largely supported by the workings of the prefrontal cortex, which is additionally home to abundant glucocorticoid receptors, executive functioning is largely influenced by the experience of stress (187; 220, 222).

Particularly, the effect of cortisol on working memory performance is pronounced (266, 267). Working memory is viewed as a division of executive functioning wherein information is ‘held’ and actively worked upon to achieve a specific outcome (267). As with memory retrieval, an inverted U-shaped relationship has been observed in relation to cortisol and working memory (124, 268). Ennis et al. (269) found that higher waking cortisol levels were significantly associated with better working memory (this association was not found for episodic memory or processing speed). Additionally, sex appears to have a modulatory effect on working memory, cortisol and stress. In one study by Schoofs et al. (270), men, but not women were found to have improved working memory performance due to stress induced by the TSST. In a meta-analysis by Shields et al. (235), the opposite finding was true: men appeared more vulnerable to the effects of stress and cortisol on working memory performance than their female counterparts. This same meta-analysis found that working memory was further modulated by time between cortisol administration and assessment, with a delay impairing performance further. This contrasts earlier meta-analyses findings which found cortisol to decrease and enhance performance after delay (235).

Finally, a broad range of cognitive functioning appears to be affected by high cortisol levels, including short term and long-term verbal memory, learning, and attention in older adults (113). Wu and Yan (258) as well as Shields et al. (267) have further explored the range of executive dysfunction associated with high chronic stress as measured by hair and salivary cortisol levels. This includes impaired spatial memory performance, poor hand-eye coordination as well as poor impulsivity control and accuracy during arithmetic tasks. Cognitive flexibility and mental rotation have additionally been identified as cognitive skills vulnerable to the effects of cortisol (247).

## Implications and clinical applicability for endocrinologists

On reflection of the information covered in this review, there are two main points that we wish to raise in terms of clinical applicability for endocrinologists. The first of which relates to the utility of cortisol measurements and the second of which relates to awareness of the psychological, cognitive, and behavioural aspects of endocrine-related disorders.

Reviewing the existing body of cortisol-related literature has demonstrated the complexity of this biological marker of HPA axis dysregulation (different samples used to determine cortisol concentrations, the timings of when the samples are taken, varying methods of measurement, storage techniques, and a range of lifestyle and biological factors that affect cortisol concentrations, etc.). What is clear though, is that a single-morning cortisol sample provides very limited predictability of the diurnal secretion. Additionally, sex and age jointly determine the 24 hour secretory profile of cortisol. In terms of diagnostic considerations, these tenuous physiological alterations bear little relevance in diagnosing major endocrine disturbances as in the case of Cushing’s disease and conditions of adrenal failure. To aid the diagnosis of such conditions, the Endocrine Societies provide clear guidelines in this regard.

The second point we wish to raise relates to raising awareness of the psychological, cognitive, and behavioural aspects of HPA axis dysfunction and endocrine-related disorders. In addition to diagnosing and treating the causes of hypercortisolaemia and adrenal insufficiency, endocrinologists should be aware of the emotional, behavioural, and cognitive symptoms often associated with these conditions (179, 185). Furthermore, future studies in this field should aim to explore further if and how longitudinal changes associated with these endocrine disorders affect (subclinical and clinical) psychopathology.

Given the links between endocrine functioning and the associations with and effects on cognitive and emotional wellbeing, endocrinologists should enquire from their patients whether they have past or current mental health difficulties. They could then consider referrals to therapy and/or psychiatric services for assessment, psychoeducation, and management. The provision of biologically-informed psychoeducation to patients about the negative impact of stress on physiological, psychological and cognitive systems may allow for appropriate intervention and cortical inhibition of fear-based responses to non-threatening stimuli. Early intervention may help patients to manage stress levels and mitigate potential adverse effects of cortisol on the body and the brain. Treatment is not just important for short-term mental health (by preventing transition to chronic anxiety and depressive symptoms, for example), it may also be key to maintaining long-term brain health (serotonin depletion and hippocampal degeneration are likely to be affected by and also have an impact on stress levels, mood, as in the case of depression, and cognitive functioning). As we get older, the impact of stress can become more severe because the ageing brain does not recover from stress as well as when we are younger. Therefore, if an individual’s stress levels are interfering with daily functioning, helpful recommendations would include seeking therapy and professional help sooner rather than later.

Stressful events are an inevitable occurrence in daily life and our ability to overcome challenges promotes a sense of success and achievement. While it is not possible, nor a helpful or realistic aim to avoid stressors completely, humans have the capacity to control their perception of and response to stressors. In this way, maladaptive cognitive appraisals, unhelpful thoughts or unchecked beliefs about the threatening nature of potential stressors may lead to an excessive physiological stress response and cortisol dysfunction. Modern day approaches to maintaining good health include identifying and addressing modifiable risk factors. Stress is a modifiable risk factor and there is increasing evidence that effectively managing stress levels has numerous benefits for psychological and physical health.

## Conclusion

The scientific stress literature is vast; it covers different theories of the impact of stress, how stress is responded to and coped with by animals and humans, effects of stress on the body and the brain, and the different contributors of stress in terms of acute stress and that which is prolonged and enduring. As a result of these complexities, the literature contains heterogeneous findings about the causes, nature, and subsequent effects of stress. This review has summarised and integrated literature on the relationships between physiological and psychosocial stress, the stress hormone cortisol, as controlled by the HPA axis, and the effects of stress on cognitive functioning.

The challenge of this review has been to draw concrete conclusions about the decades of research that have been conducted in this field. One of the main reasons for this difficulty is that the majority of stress-related research has been conducted at a group level. What is clear from the literature is that there is great individual variation in the exposure, reaction, experience, perception, and coping mechanisms of stress. These variations determine how stress affects the brain and what this then means for individuals’ vulnerability to disease.

Further contributors to the individual profile of stress response include genetic predisposition, sex, age at time of stressor occurrence, life history, personality traits, sociocultural environment, psychological resilience and coping mechanisms. Additionally, many studies have examined the impact of stress at particular time points, often relying on historical recollections of experiences. This method fails to take into account the significance of *when* adverse life events occur in relation to (brain) developmental stage. The importance of lifespan cannot be overlooked. To address the gaps in current knowledge, longitudinal studies that include a range of life stages need to be conducted. Relatedly, in older adults, elevated cortisol levels and the increased vulnerability of the brain raise further concerns about the negative impact of stress and the need for relevant interventions and stress management skills and support.

Lastly, this review has provided suggestions of clinical applicability for endocrinologists who are uniquely placed to measure outcomes related to endocrine, nervous and immune system functioning and identify areas of intervention. The key point in this regard is the importance of awareness of the psychological, cognitive, and behavioural aspects of HPA axis dysfunction and endocrine-related disorders, particularly as such symptoms may be the only manifestations of a potentially life-threatening endocrine disorder. Awareness of these aspects also lends itself to potential psychological and behavioural interventions that may provide an additional layer of support.

## Author contributions

Review written by KJ, JS, NS and MC. All authors contributed to the article and approved the submitted version.
